# Serum Alkalinization Affects Elimination of Flecainide in Chronic Toxicity: A Case Report

**DOI:** 10.1155/crcc/6227418

**Published:** 2025-11-14

**Authors:** Rafael Lima, Sydney Schacht, Alan Hyslop, Mary Wermuth

**Affiliations:** ^1^ Division of Medical Toxicology, Department of Emergency Medicine, Indiana University, Indianapolis, Indiana, USA, indiana.edu; ^2^ Department of Pulmonary Medicine, The Christ Hospital, Cincinnati, Ohio, USA, thechristhospital.com; ^3^ Division of Pulmonary, Critical Care, Sleep and Occupational Medicine, Indiana University School of Medicine, Indianapolis, Indiana, USA, indiana.edu

**Keywords:** alkalinization, case report, drug elimination, flecainide, sodium bicarbonate

## Abstract

**Background:**

Supratherapeutic flecainide concentrations may result in wide complex cardiac dysrhythmias, which are normally treated with hypertonic sodium bicarbonate therapy. Previous cases have suggested that in acute toxicity, serum alkalinization may impair the elimination of flecainide.

**Case Summary:**

We present a single case of chronic flecainide toxicity. A 69‐year‐old patient began taking oral flecainide 1 month prior and developed recurrent wide complex tachycardia (WCT) that was refractory to treatment with sodium bicarbonate and repeated defibrillations. Further arrhythmias stopped after the resolution of alkalosis and treatment with lidocaine. Serum flecainide concentrations were notable for an apparent rise from initial levels following serum alkalinization.

**Discussion:**

Medication interactions and pharmacodynamic testing could not account for increasing serum flecainide concentrations following treatment. No evidence of supratherapeutic ingestion was identified. Tissue redistribution as a result of serum alkalinization likely contributed to impaired elimination in a patient with chronic flecainide toxicity.

**Conclusions:**

Serum alkalinization from sodium bicarbonate administration has implications in the length of stay and need for adjunctive therapies in the treatment of flecainide toxicity.

## 1. Introduction

Flecainide is a class IC antiarrhythmic medication that in supratherapeutic concentrations can result in cardiac dysrhythmias through its action on cardiac voltage‐gated sodium channels (VGSCs). Flecainide has a narrow therapeutic index, and patients at risk for toxicity typically have hepatic or renal insufficiency. Metabolization occurs in the liver with oxidation producing active and inactive metabolites. As such, patients taking flecainide with hepatic impairment are at risk for supratherapeutic serum concentrations of the drug [1]. Most flecainide excretion occurs via the urine, with up to 30% eliminated as unchanged drug [2]. Patients taking oral flecainide with renal impairment have shown prolonged clearance and elimination half‐lives [3].

Hypertonic sodium bicarbonate therapy is typically effective at reversing the QRS prolonging effect of flecainide [4]. An important observed side effect of sodium bicarbonate administration is the alkalinization of the serum and urine. Studies on healthy volunteers taking therapeutic doses of flecainide have demonstrated an inversely proportional relationship between urine pH and urinary elimination of flecainide [5]. As is typical in most drug overdoses, toxicokinetics do not reliably follow pharmacokinetics in therapeutic ingestions, but this phenomenon of urinary elimination has also been observed in acute overdoses of flecainide [6]. We present a case of chronic flecainide toxicity where serum alkalinization was temporally associated with increased serum flecainide concentrations.

## 2. Case Presentation

**Figure 1:**
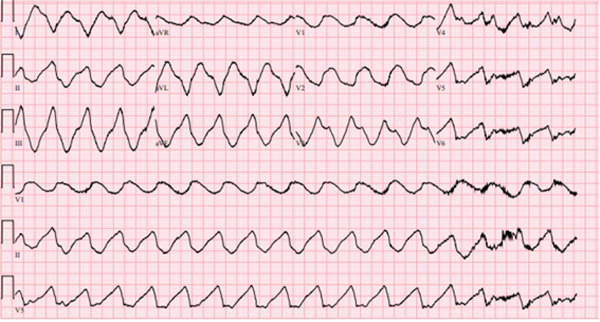
Initial electrocardiogram of the patient showing a wide‐complex arrhythmia.

**Figure 2:**
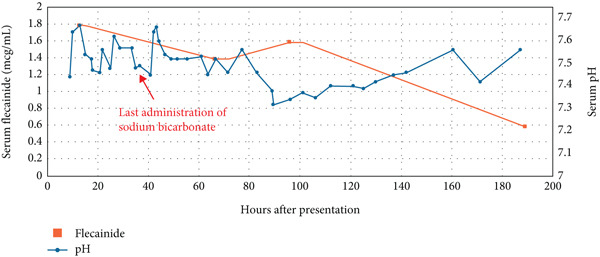
Serum flecainide concentrations obtained from the patient (represented by squares) plotted against serum pH (represented by circles) over the first 200 h of their hospital admission. Overlaid text shows the timing of the last administration of sodium bicarbonate.

A 69‐year‐old female patient with a history of atrial fibrillation and atrial flutter presented to the emergency department with lower extremity swelling and progressive orthopnea. She began taking flecainide (50 mg twice daily initially for the first week, increased to 100 mg twice daily) 1 month prior. Eight hours after presentation, she became unresponsive with a wide complex tachycardia (WCT) on electrocardiogram (QTc 794, QRS > 200 ms) (Figure 1). She was defibrillated, then went into pulseless electrical activity, for which chest compressions were started and 1 mg epinephrine was administered. She was intubated after return of spontaneous circulation 6 min later. Laboratory workup after resuscitation was significant for initial alkalosis (pH 7.64), thought to be metabolic due to bicarbonate retention and hypokalemia (2.7 mEq/L). Sodium bicarbonate was not administered during this initial arrest. Renal (creatinine 1.04 mg/dL) and hepatic function were at the patient′s baseline. The patient′s hospital course was complicated by multiple subsequent episodes of WCT, which were treated with epinephrine, multiple defibrillations, and a temporary transvenous pacemaker. Administration of 8.4% sodium bicarbonate, acetazolamide, amiodarone infusion, and 3% sodium chloride doses was unsuccessful in preventing recurrent dysrhythmias. Acetazolamide and hypertonic saline were administered intermittently and concurrently until Hour 29 into the patient′s hospital course. The last dose of sodium bicarbonate was given at Hour 37. A flecainide concentration obtained 12 h after presentation was 1.78 mcg/mL (therapeutic 0.2–1.0 mcg/mL). Subsequent flecainide levels were notable for an apparent rise after an initial decrease (Figure 2). Arterial blood gases demonstrated alkalosis (pH > 7.45) up to 89 h after hospitalization. Dysrhythmias terminated after treatment with 1 mg/kg lidocaine bolus and infusion at Hour 45, but continuous infusion was discontinued due to supratherapeutic levels of lidocaine (7.2 mcg/mL). No further doses of acetazolamide, hypertonic saline, or sodium bicarbonate were given after lidocaine administration. The patient was extubated on Day 6, the pacemaker was removed on Day 12, and she was discharged on Day 15 to acute rehabilitation. CYP2D6 pharmacogenomic testing showed normal expression of the related genes and predicted normal metabolization activity.

## 3. Discussion

Sodium bicarbonate therapy is typically used to treat widened QRS arrhythmias that occur because of medication‐induced sodium channel blockade [7]. As previously described, this therapy will alkalinize the serum and the urine, and previous reports have suggested that serum alkalinization in acute ingestions may prolong flecainide elimination [6]. In this case, an increase in serum flecainide concentrations occurred following prolonged alkalinization. Several causes for this were investigated but ultimately did not explain this observation. First, the patient described above had no evidence of decreased renal function or hepatic function to account for her prolonged flecainide elimination. Second, a review of her medication list did not reveal a drug–drug interaction that would account for a decrease in flecainide elimination; specifically, the patient did not appear to be taking other medications that impact CYP2D6 function. Third, the patient′s history did not suggest a supratherapeutic ingestion. Finally, pharmacogenomic testing did not reveal a deficiency in drug metabolization.

One explanation may relate to the redistribution of the drug from tissue into the serum. Previous reports have shown a relationship between serum and urinary pH and the amount of flecainide excreted in the urine [5, 6]. Between pH 7.7 and 7.3, the lipophilicity of flecainide drops by 2.5‐fold, with a corresponding increase in the water solubility of the same amount. Although the cause of the patient′s initial metabolic alkalosis was not determined, treatment with hypertonic sodium bicarbonate likely prolonged serum alkalinization. We propose that subsequent acidification of the serum may have been responsible for a significant shift in the drug from the lipid compartments to the hydrophilic tissues. Acetazolamide and hypertonic saline therapy may have had some contribution to serum acidification, but these therapies were stopped before the last dose of sodium bicarbonate. We suspect the withholding of alkalinizing therapy ultimately led to a return to physiologic pH.

While our case should not discourage the use of sodium bicarbonate in the treatment of sodium channel blocking agents such as flecainide, it does have implications for patient care, as drug compartment shifts may impair elimination that could ultimately increase intensive care unit and hospital length of stay. There is a potential for toxicity to be exacerbated and prolonged, as evidenced by the apparent rise in serum flecainide concentrations after serum alkalinization. It also raises the need for and study of adjunctive therapies. Hypertonic saline can aid in overcoming cardiac sodium channel blockade and does not cause the same degree of alkalinization as sodium bicarbonate. Data on its use in flecainide toxicity is limited [8]. Lidocaine has also been used in treating cardiovascular toxicity from various xenobiotic overdoses due to its mechanism of binding and dissociating quickly from the inactive conformation of VGSCs [9], and this case similarly showed success in terminating recurrence of WCT. This represents a potential treatment that would avoid the adverse indirect effects brought by sodium bicarbonate. It would also represent an additional opportunity for the study of this therapy in the treatment of flecainide toxicity.

## 4. Conclusion

This case illustrates prolonged elimination of flecainide during sustained alkalosis and redistribution effects during return to physiologic pH. It suggests that the conventional therapy of sodium bicarbonate for WCTs may introduce increases in the duration of intensive care needs and the potential for exacerbation of toxicity. While causality cannot be proven from a single case, this temporal association provides a basis for further study.

## Consent

Patient consent was obtained.

## Conflicts of Interest

The authors declare no conflicts of interest.

## Funding

No funding was received for this manuscript.

## Data Availability

The data that support the findings of this study are available on request from the corresponding author. The data are not publicly available due to privacy or ethical restrictions.
